# Retraction: The RNA-Binding Protein KSRP Promotes Decay of β-Catenin mRNA and Is Inactivated by PI3K-AKT Signaling

**DOI:** 10.1371/journal.pbio.1002314

**Published:** 2015-11-16

**Authors:** Roberto Gherzi, Michele Trabucchi, Marco Ponassi, Tina Ruggiero, Giorgio Corte, Christoph Moroni, Ching-Yi Chen, Khalid S Khabar, Jens S Andersen, Paola Briata

The authors and editors retract this publication following an investigation into concerns around the data presented in the figures that were brought to the editors’ attention. The text below has been agreed by the editors and all authors, except for G. Corte (deceased).

There were concerns raised about all of the figures. Serious issues were detected in figures 6, 7 and supplementary figures S1 and S3, including duplicated gel bands within and between these figures.

An institutional inquiry has been undertaken at the IRCCS AOU San Martino-IST in Genova (formerly IST) where Dr Gherzi undertook this research and a number of the figure anomalies have been verified by the institutional committee. Dr Gherzi would like to note specifically that his co-authors (MT, MP, TR, GC, CM, CYC, KK, JSA & PB) were not involved in the preparation of these figures. An explanation of inadvertent error was given for some of the issues identified, while for two issues, a satisfactory explanation could not be provided. Dr Gherzi assumes full sole responsibility for the errors leading to these anomalies and apologizes.

The authors maintain that the conclusions of the published paper remain valid. However, the detected errors in the published figures undermine editorial confidence in the results presented in the study. Dr Gherzi would like to note that he has sent replicate autoradiograms for each experiment to the institutional inquiring committee. However, the editors, having seen this data, could not confidently validate what was provided. Given the serious concerns that remain about the validity of some of the published data presented in this paper, the authors and editors are issuing a retraction. All authors have agreed to the retraction of this paper (except GC, for reasons noted above) and deeply regret any inconvenience this publication has caused for others.
